# Myocardial Function Prediction After Coronary Artery Bypass Grafting Using MRI Radiomic Features and Machine Learning Algorithms

**DOI:** 10.1007/s10278-022-00681-0

**Published:** 2022-08-22

**Authors:** Fatemeh Arian, Mehdi Amini, Shayan Mostafaei, Kiara Rezaei Kalantari, Atlas Haddadi Avval, Zahra Shahbazi, Kianosh Kasani, Ahmad Bitarafan Rajabi, Saikat Chatterjee, Mehrdad Oveisi, Isaac Shiri, Habib Zaidi

**Affiliations:** 1 Department of Medical Physics, School of Medicine, Iran University of Medical Sciences, Tehran, Iran; 2 Division of Nuclear Medicine and Molecular Imaging, Geneva University Hospital, Geneva 4, CH-1211 Switzerland; 3 Division of Clinical Geriatrics, Department of Neurobiology, Care Sciences and Society, Karolinska Institutet, Stockholm, Sweden; 4 Rajaie Cardiovascular Medical and Research Center, Iran University of Medical Science, Tehran, Iran; 5 School of Medicine, Mashhad University of Medical Sciences, Mashhad, Iran; 6 Department of Biostatistics, School of Health, Kermanshah University of Medical Sciences, Kermanshah, Iran; 7 School of Electrical Engineering and Computer Science, KTH Royal Institute of Technology, Brinellvägen 8, Stockholm, Sweden; 8 Comprehensive Cancer Centre, School of Cancer & Pharmaceutical Sciences, Faculty of Life Sciences & Medicine, Kings College London, London, UK; 9 Geneva University Neurocenter, Geneva University, Geneva, Switzerland; 10 Department of Nuclear Medicine and Molecular Imaging, University of Groningen, University Medical Center Groningen, Groningen, Netherlands; 11 Department of Nuclear Medicine, University of Southern Denmark, Odense, Denmark; 12 Echocardiography Research Center, Rajaie Cardiovascular Medical and Research Center, Iran University of Medical Sciences, Tehran, Iran; 13 Cardiovascular interventional research center, Rajaie Cardiovascular Medical and Research Center, Iran University of Medical Sciences, Tehran, Iran; 14 Cardio-Oncology Research Center, Rajaei Cardiovascular Medical and Research Center, Iran University of Medical Sciences, Tehran, Iran; 15 Department of Computer Science, University of British Columbia, Vancouver BC, Canada

**Keywords:** Cardiac MRI, Radiomics, Machine learning, Coronary artery bypass grafting

## Abstract

The main aim of the present study was to predict myocardial function improvement in cardiac MR (LGE-CMR) images in patients after coronary artery bypass grafting (CABG) using radiomics and machine learning algorithms. Altogether, 43 patients who had visible scars on short-axis LGE-CMR images and were candidates for CABG surgery were selected and enrolled in this study. MR imaging was performed preoperatively using a 1.5-T MRI scanner. All images were segmented by two expert radiologists (in consensus). Prior to extraction of radiomics features, all MR images were resampled to an isotropic voxel size of 1.8 × 1.8 × 1.8 mm^3^. Subsequently, intensities were quantized to 64 discretized gray levels and a total of 93 features were extracted. The applied algorithms included a smoothly clipped absolute deviation (SCAD)–penalized support vector machine (SVM) and the recursive partitioning (RP) algorithm as a robust classifier for binary classification in this high-dimensional and non-sparse data. All models were validated with repeated fivefold cross-validation and 10,000 bootstrapping resamples. Ten and seven features were selected with SCAD-penalized SVM and RP algorithm, respectively, for CABG responder/non-responder classification. Considering univariate analysis, the GLSZM gray-level non-uniformity-normalized feature achieved the best performance (AUC: 0.62, 95% CI: 0.53–0.76) with SCAD-penalized SVM. Regarding multivariable modeling, SCAD-penalized SVM obtained an AUC of 0.784 (95% CI: 0.64–0.92), whereas the RP algorithm achieved an AUC of 0.654 (95% CI: 0.50–0.82). In conclusion, different radiomics texture features alone or combined in multivariate analysis using machine learning algorithms provide prognostic information regarding myocardial function in patients after CABG.

## Introduction

According to the World Health Organization, cardiovascular diseases (CVDs) hold the title of the highest prevalent noncontagious diseases worldwide [1]. Estimates show considerably high mortality of CVD mostly happening in underdeveloped and developing countries [1, 2]. Coronary artery disease (CAD), often linked to atherosclerosis and/or aggregation of the plaque in the arteries, is the most common type of CVD [3].

Different approaches were developed for CAD treatment including pharmaceutical treatments, invasive cardiac catheterization methods (percutaneous coronary intervention (PCI)), and fully surgical solutions like coronary artery bypass grafting (CABG) [4]. For patients with severe conditions such as reduced cardiac function due to left main artery disease [5], diabetic patients [6], and patients suffering from multi-vessel disease [7], CABG is the primary treatment choice. Moreover, studies have demonstrated that CABG leads to decreased incidence of complications, including harsh cardiac and cerebrovascular illnesses, compared to PCIs [8, 9]. Hence, CABG remains the most effective treatment for patients with severe CAD [10–12]. However, CABG is an intricate, costly, and invasive procedure often coming with risks and post-operational complications (e.g., myocardial infarction (MI), cardiac stroke, graft blockage, renal dysfunction, and inflammation) [13, 14]. To extend the improvements in outcome, researchers and clinicians are constantly looking for new effective biomarkers to predict patients’ response to CABG [4, 15]. So far, the investigated biomarkers include serum and genetic biomarkers (e.g., protein biomarkers, adhesion molecule biomarkers, cytokine biomarkers, and coagulation cascade biomarkers), which are costly and invasive [4, 15].

Various medical imaging modalities, including computed tomography (CT), positron emission tomography (PET), and single-photon emission computed tomography (SPECT), can be effective for assessing and diagnosing CADs and their complications [16]. In addition to these methods, cardiac magnetic resonance imaging with late gadolinium enhancement (LGE-CMR) is also an appropriate diagnostic method for assessing myocardial function as it depicts the scar tissue developed from MI [17, 18]. Furthermore, it can be a decent predictor of a patient’s clinical outcomes [17, 19, 20]. A number of studies have shown that LGE-CMR reveals the scar region with the highest sensitivity and specificity among all methods due to its high spatial resolution [21]. However, subjective biomarkers face a lack of reproducibility since they drastically rely on the interpreter.

Radiomics can be terminologically defined as the extraction of quantitative data, such as shape, intensity, histogram, and texture features from medical images, creating a suitable feature set for the analysis of the hidden patterns in images using data mining and machine learning algorithms [22–25]. The prognostic potential of CMR radiomics and their practicality in clinical applications have been reported in multiple studies [26]. Raisi-Estabragh et al. [27] reviewed studies reporting on CMR radiomics, particularly for clinicians while discussing obstacles and shedding light on the route for further investigation. They confirmed the high potential of CMR radiomics in changing our approach to define image phenotypes, ultimately improving diagnostic accuracy, treatment choice, and prognosis. Larroza et al. [28] discriminated chronic (occurring > 6 months before imaging) and acute (occurring within 1 week) MI using radiomic features of LGE-CMR images and fed them into machine learning techniques. Avard et al. [29] investigated the potential of Cine-CMR radiomics for differentiating MI from normal tissue. Using a dataset comprising 72 patients, they achieved an AUC and accuracy of 0.93 and 0.86, when using logistic regression, and 0.92 and 0.85 for SVM, respectively. In this proof-of-concept study, we assess the potential of radiomic features extracted from LGE-CMR images along with machine learning algorithms to predict the effectiveness of the CABG operation. Our proposed methods help classify CAD patients into CABG responders and non-responders before performing this costly and invasive treatment.

## Materials and Methods

### Ethics Approval, Study Design, and Dataset

**Fig. 1:**
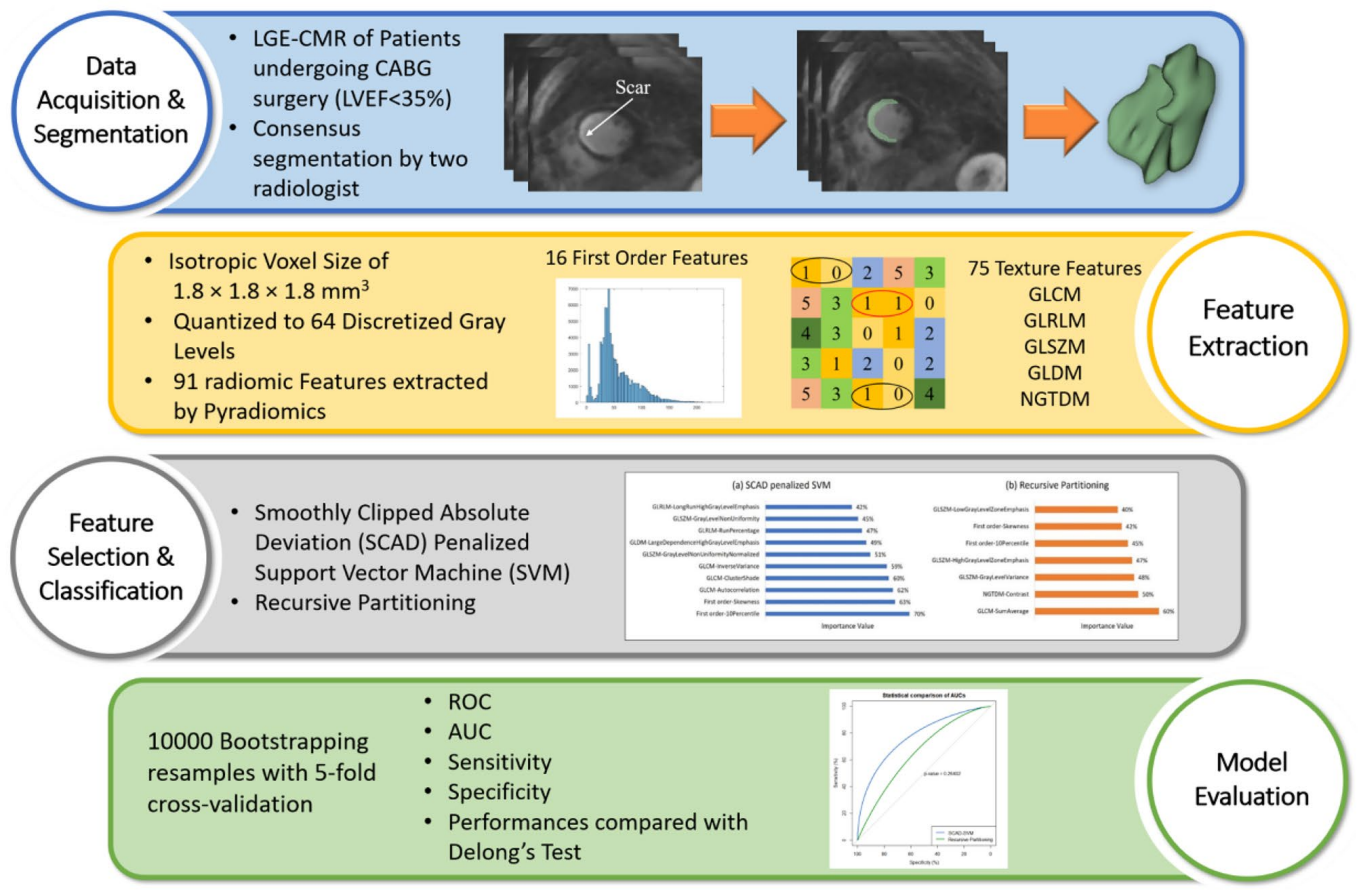
Radiomics framework adopted in the current study

The schematic framework of the study is presented in Fig. 1, which presents the different steps implemented in this study.

**Fig. 2:**
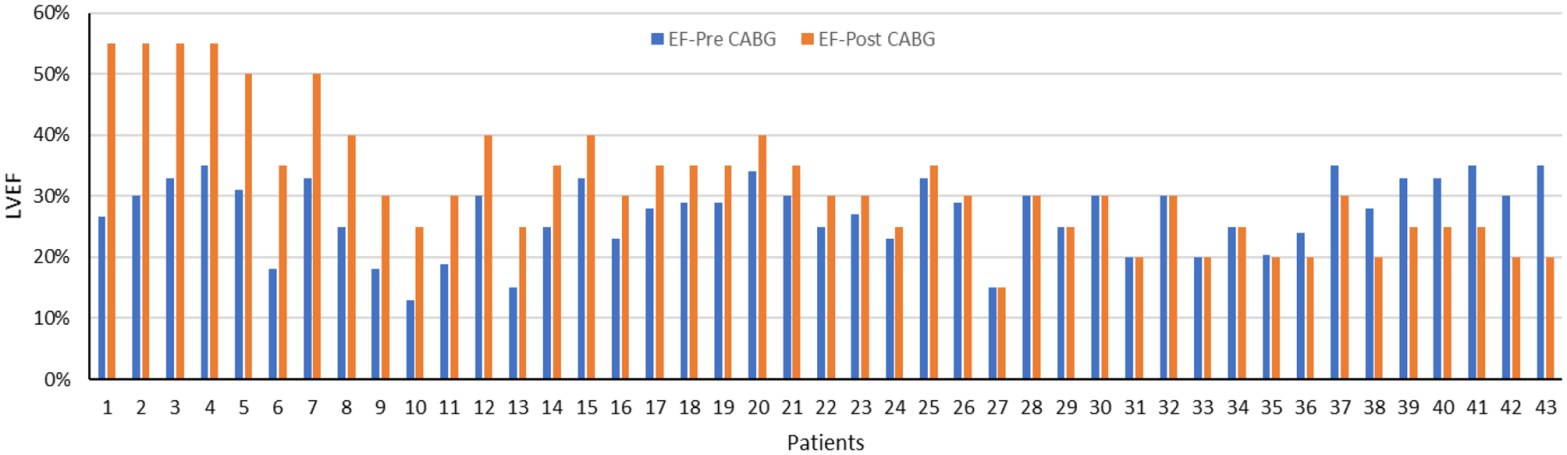
Pre- and post-CABG left ventricular ejection fraction (LVEF) of patients included in this study protocol. Patients 1–22 were responders, whereas patients 23–43 were non-responders

This retrospective study was approved by the ethics committee of Iran University of Medical Sciences (NO. IR.IUMS.FMD.REC.1398.146). Based on our inclusion criteria, 43 patients who had visible scars on short-axis LGE-CMR images and were candidates for CABG surgery (left ventricular ejection fraction (LVEF) < 35%) were selected and enrolled in this study. Table 1 shows patients’ characteristics. The LVEF of all patients was recorded before and 3 months after the CABG surgery, and patients experiencing ≥ 5% increase in LVEF (22 patients) were considered responders to CABG treatment. In addition, the pre- and post-operational LVEF of all patients is shown in Fig. 2.

**Table 1:** The demographic data of patients (total number, age, gender) are shown along with their preoperative LVEF based on each patient’s response to CABG surgical treatment

	Number of patients	Age (mean ± SD)	Gender (male/female)	LVEF % (pre/post)
Responder	22	58 ± 13	16/6	26/39
Non-responder	21	58 ± 8	18/3	28/25
Total	43	58 ± 11	34/9	27/32

### Image Acquisition and Segmentation

Patients were scanned using a 1.5-T MRI scanner (Avanto, Siemens). Ten minutes following bolus injection of 0.15 mmol/kg Gd-DTPA, LGE-CMR images were captured via a 2D Phase-Sensitive Inversion-Recovery sequence (PSIR) with TR = 683 ms, TE = 1.23 ms, flip angle = 45°, FOV = 340 × 340 mm^2^, and in-plane resolution = 1.8 × 1.8 mm^2^ in short-axis view. The slices were obtained with a 1.5-mm interval and 7-mm thickness. Abnormal regions on the images were segmented by two experienced radiologists (10 and 8 years of experience, specialized in cardiac MRI) simultaneously by consensus using the 3D-Slicer software.

### Feature Extraction

To generate rotationally invariant texture features prior to the extraction of radiomic features, all MR images were resampled to an isotropic voxel size of 1.8 × 1.8 × 1.8 mm^3^ [30]. Subsequently, intensities were quantized to 64 discretized gray levels to make the calculation of the features feasible [30]. A total of 91 features were extracted from each image using the Pyradiomics library [31]. Our feature set comprised 16 first-order features describing the distribution of intensities without considering neighboring voxels and 75 higher-order features (extracted from gray-level co-occurrence matrix (GLCM), gray-level size zone matrix (GLSZM), gray-level run length matrix (GLRLM), neighboring gray tone difference matrix (NGTDM), gray-level dependence matrix (GLDM) matrices) reflecting the textural information of the segmented areas. The features are listed in a more detailed manner in Table 2. The features extracted utilizing Pyradiomics are standardized in accordance with the Image Biomarker Standardization Initiative (IBSI) reference manuals [30].

**Table 2:** List of radiomics features extracted by Pyradiomics in this study, for first-, second, and high-order features. GLCM: Gray-level co-occurrence matrix, GLSZM: gray-level size zone matrix, GLRLM: gray-level run length matrix, NGTDM: neighboring gray tone difference matrix, GLDM: gray-level dependence matrix

First order	GLCM	GLSZM
Interquartile range	Joint average	Gray-level variance
Skewness	Sum average	Zone variance
Uniformity	Joint entropy	Gray-level non-uniformity normalized
Median	Cluster shade	Size zone non-uniformity normalized
Energy	Maximum probability	Size zone non-uniformity
Robust mean absolute deviation	Idmn	Gray-level non-uniformity
Mean absolute deviation	Joint energy	Large area emphasis
Total energy	Contrast	Small area high gray-level emphasis
Root mean squared	Difference entropy	Zone percentage
90percentile	Inverse variance	Large area low gray-level emphasis
Entropy	Difference variance	Large area high gray-level emphasis
Range	Idn	High gray-level zone emphasis
Variance	Idm	Small area emphasis
10percentile	Correlation	Low gray-level zone emphasis
Kurtosis	Autocorrelation	Zone entropy
Mean	Sum entropy	Small area low gray-level emphasis

GLRLM	MCC	GLDM
Short-run low gray-level emphasis	Sum squares	Gray-level variance
Gray-level variance	Cluster prominence	High gray-level emphasis
Low gray-level run emphasis	Imc2	Dependence entropy
Gray-level non-uniformity normalized	Imc1	Dependence non-uniformity
Run variance	Difference average	Gray-level non-uniformity
Gray-level non-uniformity	Id	Small dependence emphasis
Long-run emphasis	Cluster tendency	Small dependence high gray-level emphasis
Short-run high gray-level emphasis	**NGTDM**	Dependence non-uniformity normalized
Run length non-uniformity	Coarseness	Large dependence emphasis
Short-run emphasis	Complexity	Large dependence low gray-level emphasis
Long-run high gray-level emphasis	Strength	Dependence variance
Run percentage	Contrast	Large dependence high gray-level emphasis
Long-run low gray-level emphasis	Busyness	Small dependence low gray-level emphasis
Run entropy		Low gray-level emphasis
High gray-level run emphasis		
Run length non-uniformity normalized		

### Univariate and Multivariable Analyses

In this study, we utilized two embedded methods to simultaneously select the optimum feature set and construct classifier models. The methods included smoothly clipped absolute deviation (SCAD)–penalized support vector machine (SVM) (“penalized SVM” R package) [32] and recursive partitioning (RP) for (“rpart” R package) [33] algorithms for binary classification. The SCAD-SVM algorithm is a flexible and robust method providing the advantages of the SCAD penalty while at the same time avoiding sparsity limitations for non-sparse data in high-dimensional structure data [34]. In addition, the PR algorithm is a nonparametric and consistent method designed to find local low-dimensional structures in functions that have a high-dimensional global dependence [35]. The cutoff point for the selected features was identified based on the maximization Youden index. The importance value of the selected features was calculated by gain information measure. After obtaining the feature sets, we constructed univariate models using each selected feature individually in addition to the multivariable models to achieve a better insight into effective features.

All models were evaluated and validated by fivefold cross-validation with 10,000 bootstrapping resamples. As a tuning parameter in the SCAD penalty method, optimal lambda was estimated by the minimized cross-validation error rate. After fitting the SCAD-penalized SVM and RP algorithms, the area under the ROC curve (AUC), sensitivity, and specificity were calculated in order to assess the predictive power of each selected feature and the whole feature set overall. The importance value of the features was also calculated. Finally, the statistical comparison of the area under the ROC curve (AUCs) between the two algorithms was performed by DeLong’s test using the “pROC” R package [36] with a statistical significance level of 0.05.

## Results

**Fig. 3:**
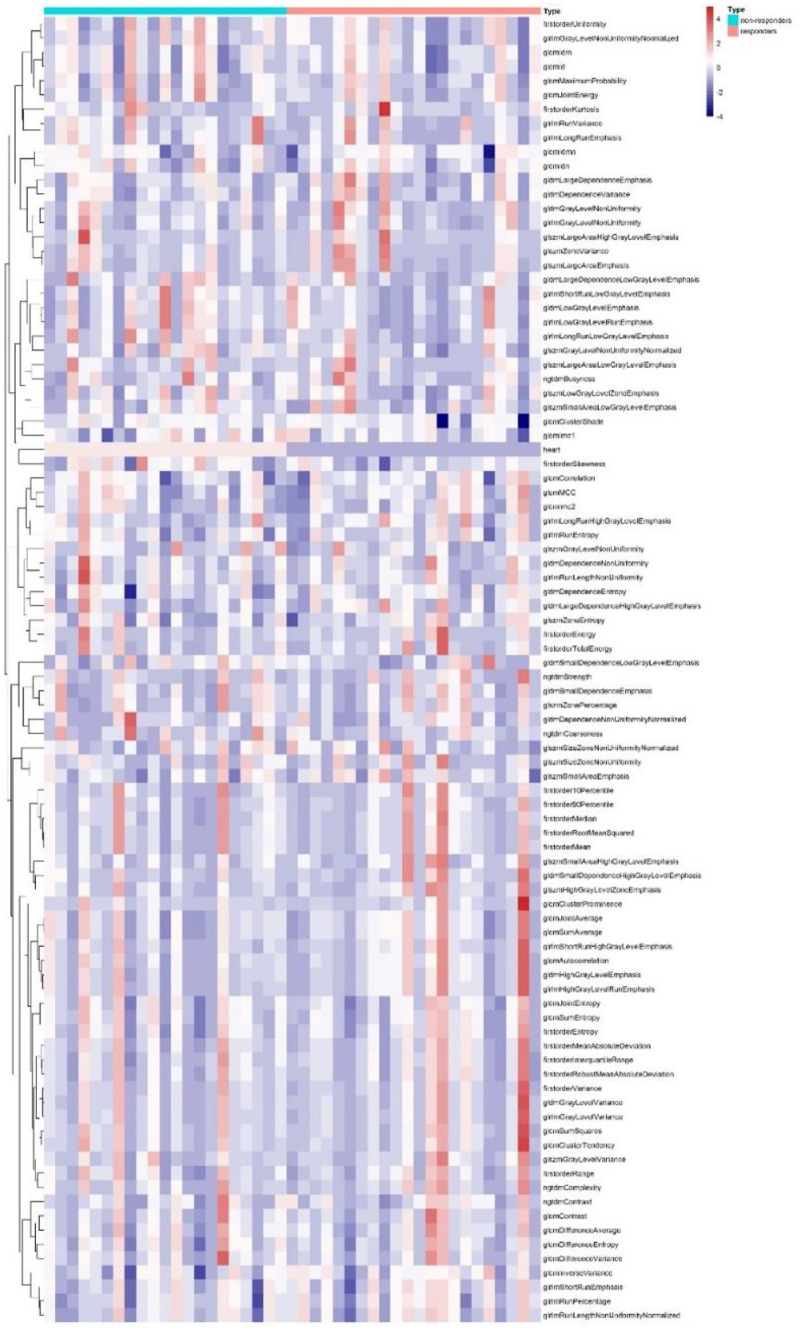
Cluster heat map of radiomic features for responder and non-responder groups

Figure 3 represents an unsupervised cluster heat map of radiomic features in two groups, which did not show any specific cluster class in dataset.

**Fig. 4:**
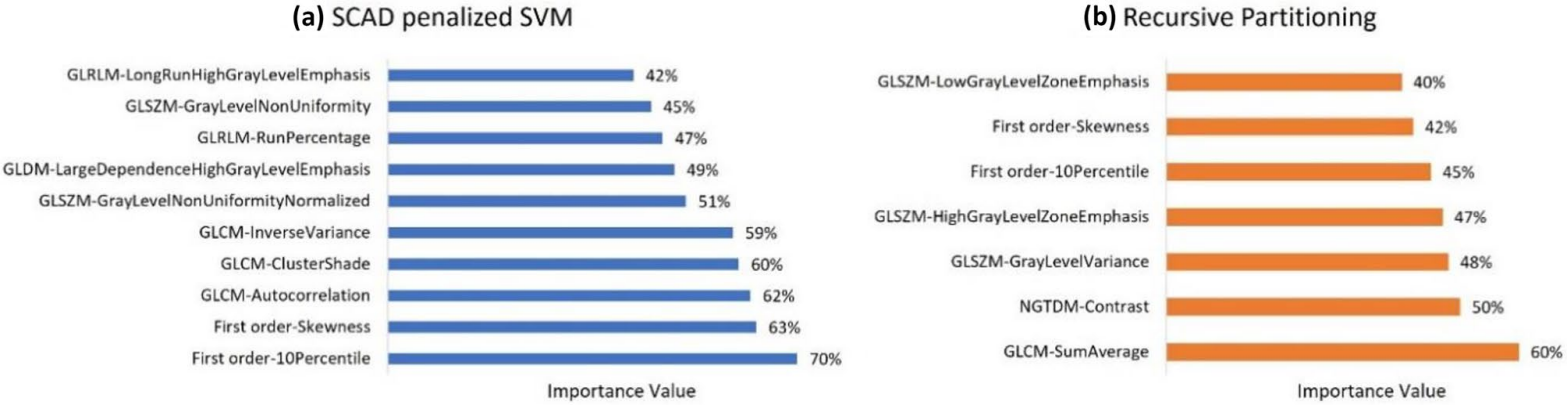
Importance value of the selected features using **a** SCAD penalized SVM and **b** recursive partitioning algorithms

Figure 4 shows selected features and their importance value using (a) SCAD-penalized SVM and (b) RP algorithms. As can be seen, 10 features were selected with SCAD-penalized SVM (importance value ranging from 42 to 70%), and 7 features selected by the RP (importance values ranging from 40 to 60%) algorithm for CABG responder/non-responder classification. The selected feature sets were a combination of first-order and texture features. First-order 10 percentile (importance value: 70%) and GLCM sum average (importance value: 60%) features were the most important features in SCAD-penalized SVM and RP algorithms, respectively.

**Fig. 5:**
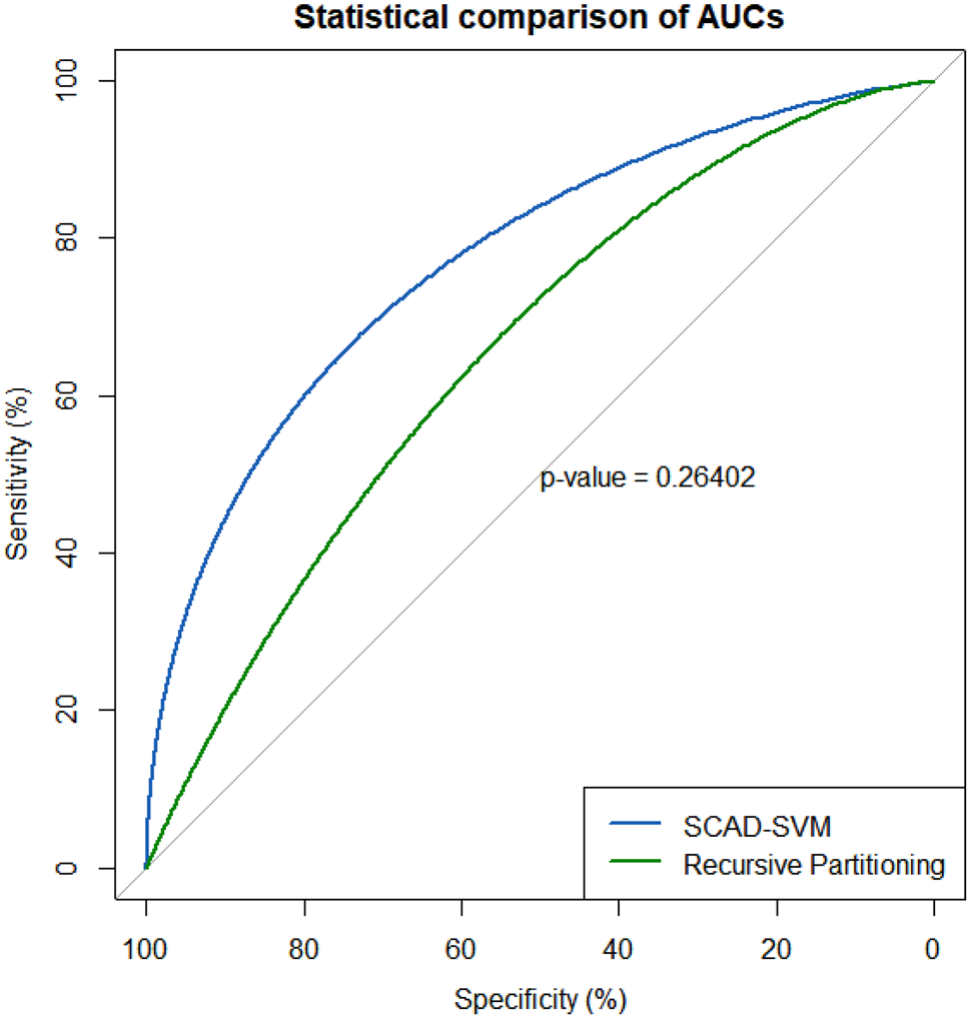
ROC curves for SCAD-SVM and RP classifiers. The statistical comparison of the two ROC curves with 10,000 bootstrapping resamples showed no statistically significant difference (*p* value = 0.264)

Table 3 shows the area under the ROC curve (AUC) with 95% confidence intervals, sensitivity, and specificity with standard deviation for both univariate analysis and multivariable/overall models. Considering univariate analysis, GLSZM gray-level non-uniformity-normalized (AUC = 0.62, 95% CI: 0.53–0.76) and first-order skewness (AUC = 0.59, 95% CI: 0.5–0.68) achieved the best performance using SCAD-penalized SVM and RP algorithms, respectively. Regarding multivariable modeling, SCAD-penalized SVM achieved statistically insignificant (*p* value = 0.264) higher performance (AUC = 0.784, 95% CI: 0.64–0.92) compared to the RP algorithm (AUC = 0.654, 95% CI: 0.50–0.82) (Fig. 5).

**Table 3:** The area under the ROC curve (AUC) with 95% confidence intervals, sensitivity, and specificity with standard deviation for each selected feature (univariate) and whole feature set (multivariable analysis). The “*” sign indicates significant predictive variables by univariate ROC curve analysis at the level of 0.05

Method	Selected variables	AUC (95% CI)	Sensitivity	Specificity	Overall AUC (95% CI), sensitivity (SD), specificity (SD)	DeLong’s test for comparison of two ROC curves
**SCAD-penalized SVM**	First order-10 percentile	0.55 (0.40–0.71)	0.82	0.30	0.784 (0.64–0.92), 0.591 (0.09), 0.809 (0.10),	*Z* = 1.189 (*p* value = 0.264)
	First order-skewness	0.59 (0.50–0.68)*	0.73	0.53		
	GLCM-autocorrelation	0.56 (0.40–0.71)	0.64	0.53		
	GLCM-cluster shade	0.55 (0.40–0.70)	0.77	0.43		
	GLCM-inverse variance	0.60 (0.52–0.75) *	0.68	0.57		
	GLSZM-gray-level non-uniformity normalized	0.62 (0.53–0.76) *	0.69	0.62		
	GLDM-large dependence high gray-level emphasis	0.59 (0.50–0.73) *	0.55	0.67		
	GLRLM-run percentage	0.57 (0.42–0.72)	0.46	0.76		
	GLSZM-gray-level non-uniformity	0.60 (0.50–0.74) *	0.68	0.53		
	GLRLM-long-run high gray-level emphasis	0.55 (0.39–0.70)	0.55	0.67		
**RP algorithm**	GLCM-sum average	0.56 (0.40–0.71)	0.91	0.24	0.654 (0.50–0.82), 0.727 (0.11), 0.523 (0.09),	
	NGTDM-contrast	0.56 (0.40–0.71)	0.86	0.34		
	GLSZM-gray-level variance	0.54 (0.38–0.69)	0.64	0.57		
	GLSZM-high-gray-level zone emphasis	0.54 (0.38–0.69)	0.59	0.57		
	First order-10 percentile	0.55 (0.40–0.71)	0.82	0.30		
	First order-skewness	0.59 (0.50–0.68) *	0.73	0.53		
	GLSZM-low gray-level zone emphasis	0.55 (0.39–0.70)	0.32	0.76

## Discussion

Leading to lethal conditions, such as MI, CAD is often promptly treated with pharmaceutical medications or invasive procedures, such as PCI and CABG [37]. Based on previous studies, patients who underwent CABG treatment show less postoperation complications in comparison with patients treated with PCI [8, 9], which explains the high prescription of CABG for patients with severe CAD [10, 11]. The lethal condition of CAD and the invasiveness of conventional treatments raise demand for robust diagnosis and prognostic methods to characterize the disease and predict treatment outcomes. In recent years, medicine has been remarkably influenced by the discovery of biomarkers supplying prognostic evidence and information for clinicians to predict clinical outcomes. However, compared to other fields of medicine such as oncology, fewer studies have investigated to identify biomarkers for cardiac prognosis, especially for treatment outcome prediction based on images.

Radiomics and machine learning have been recently applied to different modalities of cardiac imaging to provide diagnostic and prognostic models. In a study by Antunes et al. [38], seven patients were examined for the discrimination between normal and scarred myocardium using texture analysis of cardiac CT images. They achieved 94% accuracy for differentiating between normal and scar tissue. Shu et al. [39] examined CT-based radiomics machine learning to predict chronic myocardial ischemia in 154 patients using 378 extracted texture features. They reported an accuracy of 0.83 for the radiomics nomogram designed for the detection of myocardial ischemia. Larroza et al. [28] examined the differentiation between acute from chronic MI in 44 patients using machine learning techniques and MRI texture features. They reported sensitivity, specificity, and AUC of 0.79, 0.85, and 0.85, respectively, as the best results. Baeßlera et al. [40] suggested a model for discovering tissue change in hypertrophic cardiomyopathy (HCM) patients on CMR images without any contrast agent. Using the random forest algorithm, they could achieve a sensitivity and specificity of 91% and 93%, respectively. Another study has been designed for discriminating between hypertensive heart disease and HCM in T1 mapping. They reported an accuracy of 86.2% using SVM classifier and the selection of texture features [41].

Baessler et al. [42] investigated the combination of different texture features using multiple logistic regression models. The AUC of their models reached 0.93 and 0.92 for diagnosing large and small MI on cine MR images, respectively. Eftestøl et al. [43] concluded that texture analysis of LGE-CMR images is able to identify high- and low-risk cardiac patients and discriminate them for using ICD implantation. Their results indicated that texture analysis of LGE-CMR images includes data that can boost the capability of predicting a target up to a 0.84 specificity. Shao et al. [44] reported that a machine learning–based SVM model reached an accuracy of 0.85 with the aid of histogram and GLCM features to distinguish between dilated cardiomyopathy (DCM) patients and control groups using T1 MR images. Chen et al. [45] examined radiomic analysis in 70 patients with ST-elevation MI for the differentiation between reversible versus irreversible myocardial damage. Five texture features were extracted from contrast T1 mapping, reporting an AUC of 0.91 (*p* < 0.0001) for the differentiation between reversible and irreversible myocardial damage. Another study revealed that texture analysis is capable of diagnosing Takotsubo syndrome in 58 patients. In their study, T2-weighted MRI texture features were fed to a naïve Bayes machine learning classifier providing overall best performance with a sensitivity of 82.9% (95% CI:80–86.2), specificity of 83.7% (95% CI:75.7–92), and AUC of 0.88 (95% CI:0.83–0.92) [46].

Although the effectiveness of CABG has not been assessed by CMR radiomics before, the outcome prediction of other interventional treatments was previously investigated. Ma et al. [47] conducted a study to develop a radiomics model based on features extracted from T1-mapped CMR scans for predicting major adverse cardiac events threatening patients with acute ST elevation MI undergoing PCI. By enrolling 157 patients, their model performed well in the test set with an AUC of 0.90 and F1 score of 0.62. More comprehensive studies are needed to consider both scenarios (PCI and CABG) and compare the most predictive features and overall performance. In this study, we investigated the potential of LGE-CMR texture analysis for CABG outcome prognosis. As a proof-of-concept study, our results provide a body of evidence, asserting that texture analysis of LGE-CMR scans has the potential to characterize the underlying pathology of lesions to differentiate between responder and non-responder CABG candidate patients. We established robust multivariable classifiers providing significant predictive power (AUCs of 0.784 and 0.654 for SCAD penalized SVM and RP, respectively). By distinguishing CABG non-responder patients, they can benefit from alternative effective treatment strategies such as pharmacological treatments or be directed toward heart transplantation surgery.

One of the bright sides of radiomics analysis, in comparison with more complicated methods, such as deep learning, is its ability to identify predefined standard features that correlate well with the outcome of interest. While such interpretation may not always be straightforward, more and more studies are attempting to justify univariate outcomes by exploring possible explanations for the high performance achieved by certain features [22, 24, 48, 49]. This can also help toward offsetting probable biases in results and conclusions, which is very common in radiomics studies owing to the unavailability of large datasets and the lack of external validation. Our results also highlighted single features that are capable of significantly stratifying patients into classes. In necrosis areas, the elevated extracellular volume and the decreased speed of washout result in the accumulation of gadolinium, which produces a stronger signal in the LGE-CMR scan [50]. GLSZM gray-level non-uniformity normalized (szm-glnu-norm) from the SCAD-SVM algorithm had the highest important value. This feature calculates the distribution of zone counts over the gray levels. A higher value of this feature means that zone counts are distributed unequally over the gray levels [51]. We hypothesized that this feature reflects poor prognosis due to the presence of severely infarcted zones (zones with high gadolinium accumulation) that cannot be revived even after oxygen is supplied via surgical revascularization. The other most predictive feature was skewness in the RP algorithm. Skewness is the representative of unsymmetrical distribution of gray-level values in the region of interest. A negative skew in an image is associated with a high number of voxels with a strong signal, here indicating high gadolinium accumulation by the severely infarcted tissue. Hence, skewness is also in concordance with szm-glnu-norm representing infarcted myocardial tissue showing poor prognosis.

Regarding machine learning algorithms, we utilized two robust/consistent classifiers with built-in embedded feature selections. Embedded feature selection methods identify the subset of features that optimize the performance of the desired machine learning algorithm through considering interactions of features and simultaneously keeping computational costs reasonably low (40). Although the SCAD-SVM model achieved higher AUC compared to the RP algorithm (0.784 vs 0.654), their difference was not significant based on the comparison of their AUCs using DeLong’s test. Moreover, both models were significantly predictive considering ROC curve analysis at a level of 0.05. Putting together the positive results of both multivariable models and certain single features, we can strengthen the evidence that radiomic features extracted from LGE-CMR scans of CAD patients can predict CABG outcome regardless of the feature selection and machine learning algorithm. However, the performance of the model can be optimized by selecting the most appropriate model. Future studies might explore more advanced and complex machine learning models and their integration with ensemble learning methods.

This preliminary study suffered from a number of limitations. To start with, the number of patients was relatively small. However, our dataset was well-balanced with respect to responder and non-responder patients. In addition, we used repeated fivefold cross-validation with 10,000 bootstrapping resamples. Overall, considering the study as a proof-of-concept, the number of patients was sufficient. Future studies should enroll larger datasets to further validate our findings and extend the model to a more robust and reproducible condition. Different studies used different criteria to assess the effectiveness of CABG surgery on myocardium functionality [52–54]. In this study, the difference between pre- and post-CABG LVEF of patients obtained with echocardiography was used as a threshold to classify them into responder and non-responder classes. The threshold of 5% increase was selected to ensure the difference is not due to the echocardiography error. Different studies use different criteria based on what data they have access to. To improve the reliability of studies of this kind, standardization is needed for the evaluation of CABG treatment outcomes.

## Conclusion

The results of this study showed that machine learning algorithms can provide useful insight into the prediction of myocardial function in patients after CABG. Multiple radiomics texture features alone or combined in the multivariable model using machine learning algorithms provide prognostic information regarding myocardial function improvement in patients after CABG.

## Data Availability

Not applicable.

## Code Availability

Open-source library including SERA and R library used in this study.
